# Organizational Resilience to Supply Chain Risks During the COVID‐19 Pandemic

**DOI:** 10.1111/1467-8551.12648

**Published:** 2022-08-29

**Authors:** Nur Baiti Ingga Wulandhari, Pawan Budhwar, Nishikant Mishra, Saeed Akbar, Quynh Do, Gavin Milligan

**Affiliations:** ^1^ Faculty of Business, Law and Politics University of Hull Hull HU6 7RX UK; ^2^ Aston Business School Aston University Birmingham B4 7ET UK; ^3^ Faculty of Management, Law and Social Sciences University of Bradford Bradford BD7 1DP UK; ^4^ Lancaster University Management School Lancaster University Lancaster LA1 4YW UK; ^5^ Green Knight Sustainability Consulting Hull UK

## Abstract

This paper aims to establish a link between aggregate organizational resilience capabilities and managerial risk perception aspects during a major global crisis. We argue that a multi‐theory perspective, dynamic capability at an organizational level and enactment theory at a managerial level allow us to better understand how the sensemaking process within managerial risk perception assists organizational resilience. We draw from in‐depth interviews with 40 managers across the UK's food industry, which has been able to display resilience during the pandemic. In sensing supply chain risks (SCRs), managers within both authority‐based and consensus‐based organizational structures utilize risk‐capture heuristics and enact actions related to effective communications, albeit at different information costs. In seizing, we found that managers adhere to distinct heuristics that are idiosyncratic to their organizational structures. Through limited horizontal communication channels, authority‐based structures adhere to rudimentary how‐to heuristics, whereas consensus‐based structures use obtainable how‐to heuristics. We contribute to the organizational resilience and dynamic capabilities literature by identifying assessment as an additional step prior to transforming, which depicts a retention process to inform future judgements. Our study presents a novel framework of organizational resilience to SCRs during equivocal environments, by providing a nuanced understanding of the construction of dynamic capabilities through sensemaking.

## Introduction

During highly uncertain and turbulent times, organizations frequently face risks and disruptions across their supply chains (Ambulkar, Blackhurst and Grawe, 2015; Brandon‐Jones *et al.*, 2014; Dubey *et al.*, 2021; Knemeyer, Zinn and Eroglu, 2009; Remko, 2020). The importance of resilience has stood out as a way to understand how organizations cope with these environments (Ambulkar, Blackhurst and Grawe, 2015; Chopra and Sodhi, 2014; Conz and Magnani, 2020; Hillmann and Guenther, 2021; Ponomarov and Holcomb, 2009; Wulandhari *et al.*, 2022). Organizations’ resilience is often associated with dynamic capabilities (Teece, Pisano and Shuen, 1997), namely their ability to adapt, integrate and reconfigure their resources to cope and further thrive in changing conditions (e.g. Ambulkar, Blackhurst and Cantor, 2016; Brusset and Teller, 2017). Most empirical studies investigate resilience by retrospective analysis, adopting theories which imply the traditional assumption of rational decision‐makers, such as the resource‐based view of the firm (RBV) (e.g. Ponomarov and Holcomb, 2009) and systems theory (e.g. Spiegler, Naim and Wikner, 2012). However, evidence shows that during uncertainties, actors often violate this assumption (Hinterhuber, 2015; Jiang and Tornikoski, 2019; Julmi, 2019; Kahneman and Tversky, 1982; Li and Ahlstrom, 2020). Thus, ‘it remains unclear what resilient organisations actually do and how organisational resilience may be achieved in practice’ (Duchek, 2020, p. 216).

Due to lack of information in the face of equivocal environments, managers are confronted by insufficient visibility of their supply chains, thus facing substantive uncertainty in the evaluation of supply chain risks (SCRs) (Dosi and Egidi, 1991). The advancement of behavioural research in organizational and supply chain research provides evidence for the importance of managerial perceptions of risks in decision‐making (Sarafan, Squire and Brandon‐Jones, 2019; Zsidisin, 2003; Zsidisin and Wagner, 2010), crucial to the development of resilience (Ambulkar, Blackhurst and Cantor, 2016). Sensemaking, defined as a ‘socio‐psychological process that occurs when individuals face discrepant cues in their environment and involves the retrospective development of a plausible mental model of the situation that facilitates information processing and decision‐making’ (Sarafan, Squire and Brandon‐Jones, 2019, p. 237), is considered useful for noting the behavioural process of individuals’ risk perceptions in resolving equivocality (Ellis, Shockley and Henry, 2011; Olcott and Oliver, 2014; Sarafan, Squire and Brandon‐Jones, 2019). Nevertheless, the literature is still in its infancy (Sarafan, Squire and Brandon‐Jones, 2019). We particularly lack knowledge about how the process of sensemaking within risk perceptions may affect the development of organizational resilience to SCRs (Tisch and Galbreath, 2018; Whiteman and Cooper, 2011).

The purpose of this study, therefore, is to develop a novel framework to advance our understanding of *how* resilience can be achieved during equivocal environments, with a particular focus on investigating the role of managerial risk perception in assisting the assembly of dynamic capabilities. We adopt an abductive approach to compare the formation of a new framework with an extant literature‐based theory (Dubois and Gadde, 2002). By taking a behavioural view of dynamic capabilities, we establish the link between aggregate organizational resilience capabilities and aspects of managerial risk perception based on heuristics and organizational structures. To develop theoretical grounding at the individual level, enactment theory has been adopted to understand the sensemaking process within managerial risk perception (Weick, 2001), and dynamic capabilities are used to explore organizational level resilience (Teece, 2007). The global crisis of COVID‐19 yields a special case of an equivocal situation that is causing massive supply chain disruption (Queiroz, Fosso Wamba and Branski, 2022; Shen and Sun, 2021) and global systemic shock (Bailey and Breslin, 2021; Ivanov, 2020; Verbeke, 2020). It provides us with a unique and rich context to study the interplay between managerial risk perception and the resilience of organizations to SCRs during a major crisis. With an analysis of 40 in‐depth interviews with managers working in the UK's food supply chains (FSCs) during the COVID‐19 global pandemic, we have developed a framework of organizational resilience to SCRs in equivocal environments.

Our proposed framework fosters a more nuanced and comprehensive understanding of how managerial sensemaking processes can assist the construction of dynamic capabilities to achieve organizational resilience to SCRs. Enactment theory elaborates our understating of the role of sensemaking within managerial risk perception in assisting the assembly of organizational dynamic capabilities (i.e. sensing through enactment, seizing through selection, assessment and transformation through retention). Furthermore, an additional step of ‘assessment’ prior to ‘transforming’ is crucial within dynamic capabilities during equivocal environments which depict the process of retention, emphasizing the importance of cause–effect relationships to enhance future judgements. Our results suggest that the dynamic capabilities approach varies within different organizational structures’ communication channels (i.e. vertical and horizontal), which serve as a platform to the production of distinct heuristics utilized within the sensemaking process. Although at different information costs, both structures (i.e. authority‐based and consensus‐based) adhere to risk‐identification heuristics in the sensing phase, to enact information and actions in the identification of risks. Due to different rules of coordination within each structure, actors follow distinct heuristics during the seizing phase to select appropriate actions: rudimentary how‐to heuristics within authority‐based structures and obtainable how‐to heuristics within consensus‐based structures. We therefore argue that both structures can stimulate resilience despite requiring different coordination practices at different levels of information costs.

## Literature review

### Dynamic capabilities of organizational resilience to supply chain risks

The association of resilience to dynamic capabilities (Teece, Pisano and Shuen, 1997) lies in its function as ‘evolutionary fitness’ (Hendry *et al.*, 2019, p. 7), which enables the creation, extension and modification of the resource base to generate long‐term success. The entailed phases of sensing, seizing and transforming (Teece, Pisano and Shuen, 1997) help organizations to manage their resources in order to cope and further thrive to match the requirements of changing conditions (Davis, Eisenhardt and Bingham, 2009). Recognizing these capabilities beyond the context of restoration and maintenance, this capability view of organizational resilience can be divided into two different categories within differing temporal contexts^1^: adaptation (i.e. ability to recover and advance organizational processes and capabilities beyond maintenance and restoration *during* and *after* disruptions) and anticipation (i.e. ability to identify potential risks to take proactive steps *before* disruptions) (Duchek, 2020) (see Table 1 for summary).

**Table 1:** Differing capability view of organizational resilience definitions

**Resilience with the capability of…**	**Author(s)/year**	**Resilience definition**
*Adaptation* (i.e. ability to recover and advance organizational processes and capabilities beyond maintenance and restoration *during* and *after* disruptions)	Tognazzo, Gubitta and Favaron (2016)	‘Organisation's capacity to adjust to challenging conditions like environmental shocks, and emerge from them strengthened and more resourceful’ (p. 772)
	Ambulkar, Blackhurst and Grawe (2015)	‘Firm's resilience to supply chain disruptions is defined as the capability to be alert to, adapt to, and quickly respond to changes brought by a supply chain disruption’ (p. 122)
	Lengnick‐Hall, Beck and Lengnick‐Hall (2011)	‘The firm's ability to effectively absorb, develop situation‐specific responses to, and ultimately engage in transformative activities to capitalize on disruptive surprises that potentially threaten organization survival’ (p. 244)
	Vogus and Sutcliffe (2007)	‘The maintenance of positive adjustment under challenging conditions such that the organization emerges from those conditions strengthened and more resourceful’ (p. 3418)
	Demmer, Vickery and Calantone (2011)	‘Ability to continually evolve and thrive over time in the face of adverse, and sometimes hostile, circumstances which naturally arise in dynamic environments’ (p. 5398)
*Anticipation* (i.e. ability to identify potential risks in order to take proactive steps *before* disruptions)	Morais‐Storz and Nguyen (2017)	‘Ability to dynamically reinvent business models and strategies as circumstances change, to continuously anticipate and adjust to changes that threaten their core earning power and to change before the need becomes desperately obvious’ (p. 96)
	Ortiz‐de‐Mandojana and Bansal (2016)	‘The incremental capacity of an organization to anticipate and adjust to the environment’ (p. 6)
	van Essen *et al.* (2015)	‘Firm's capacity to perceive, avoid, absorb, adapt to and recover from environmental conditions that could threaten their survival, is subject to similar contentions’ (p. 167)
	Boin and van Eeten (2013)	Precursor resilience ‘prevents budding problems from escalating into a full‐blown crisis or breakdown’ (p. 431)
	Somers (2009)	Resilience ‘is more than mere survival; it involves identifying potential risks and taking proactive steps (…) to ensure that an organization thrives in the face of adversity’ (p. 13)

Source: Adapted from Conz and Magnani (2020) and Duchek (2020).

The first stream explores resilience as an organization's ability to absorb shocks to maintain stability and further examines how they can engage in transformative activities to cope with unexpected events (Duchek, 2020). It conceptualizes resilience as an organization's ability to recover and survive adverse conditions, develop situation‐specific responses and ultimately engage in transformative activities (e.g. Lengnick‐Hall, Beck and Lengnick‐Hall, 2011; van Essen *et al.*, 2015). For example, Demmer, Vickery and Calantone (2011) refer to resilience as the organization's ability to continually evolve and thrive in the face of adverse and sometimes hostile circumstances, similar to what others label as ‘strategic offence’ (Limnios *et al.*, 2014) or ‘strategic resilience’ (Hamel and Vaelikangas, 2003). In general, this line of work contributes to the resilience field by providing insights into conditions for the development of resilience and its internal workings (e.g. Lengnick‐Hall, Beck and Lengnick‐Hall, 2011; Ortiz‐de‐Mandojana and Bansal, 2016).

The second stream covers resilience prior to the occurrence of a disturbance (Conz and Magnani, 2020). Researchers have conceptualized resilience as an attribute that organizations possess before an event occurs (Somers, 2009). They explore key resilience measures that firms need to develop, to sense and respond to different disturbances such as social, market, financial and operational, and further assess their organizational impacts (e.g. Abeysekara, Wang and Kuruppuarachchi, 2019; Baghersad and Zobel, 2022; Brewton *et al.*, 2010; Narasimhan and Talluri, 2009; Wagner and Bode, 2008).

Further evidence in the existing literature suggests that organizational success and growth during crises can only be achieved by combining different approaches (Alikhani, Torabi and Altay, 2021; Conz and Magnani, 2020; Duchek, 2020; Williams *et al.*, 2017). Following the procedure for developing a construct definition based on prior literature (Ambulkar, Blackhurst and Grawe, 2015; Gilliam and Voss, 2013; Raetze *et al.*, 2021), we define organizational resilience during equivocal environments based on the two‐capabilities approach (i.e. adaptation and anticipation) as: *the ability of organizations to identify potential risks in order to take proactive steps, along with the ability to recover and advance organizational processes and capabilities beyond maintenance and restoration*.

While the extant literature provides important insights into the dynamic nature of resilience (Conz and Magnani, 2020; Duchek, 2020), the context‐dependent value of dynamic capabilities should be acknowledged (Wilden *et al.*, 2013). The unprecedented crisis of COVID‐19 further emphasizes this notion by bringing ‘the importance of interdisciplinary scholarship to the forefront’ (Budhwar and Cumming, 2020, p. 1), prompting examinations of resilience through combinations of theoretical underpinnings. For instance, to bring a novel approach to resilience during COVID‐19, Ivanov (2021) presents an integrative conceptualization of resilience based on different existing frameworks. Building on the socio‐technical characteristics of digital platforms, Floetgen *et al.* (2021) develop the concept of platform ecosystem resilience in the context of COVID‐19. Modgil, Singh and Hannibal (2021) further explore the application of artificial intelligence to develop supply chain resilience to withstand extreme disruptions.

Furthermore, there is growing awareness of how COVID‐19 has exposed the vulnerability of the food industry and systems (Fan *et al.*, 2021). We argue that although the importance of food chain resilience has been continuously emphasized by prior studies (e.g. Hendry *et al.*, 2019; Leat and Revoredo‐Giha, 2013; Stone and Rahimifard, 2018), the accentuated fragility of this sector caused by COVID‐19 warrants further examination (Burgos and Ivanov, 2021). In the spirit of maintaining this momentum, we fuse the behavioural perspective with dynamic capabilities to highlight the role of managerial risk perception to further promote the understanding of resilience during the equivocal environment of COVID‐19 in the food sector.

### A behavioural view of dynamic capabilities during equivocal environments

Applications of dynamic capabilities have gained considerable scholarly attention, being commonly combined with supporting theoretical stances. For example, dynamic capabilities have been grounded in RBV (e.g. Brusset and Teller, 2017), systems thinking (e.g. Cezarino *et al.*, 2019) and, more recently, the application of artificial intelligence (e.g. Belhadi *et al.*, 2021; Modgil, Singh and Hannibal, 2021). Despite providing significant value in showcasing the importance of capabilities and resources requiring dynamic adaptation to the changing environment, most of these studies are rooted in a simplistic ‘reductionist’ worldview (Pettit, Croxton and Fiksel, 2019) (see Table 2 for summary). It implies the ‘traditional’ lens of rational decision‐makers where, according to Boudreau *et al.* (2003), several assumptions of human behaviour are involved: people are (1) not the main phenomenon under study; (2) deterministic in their behaviour; (3) independent; (4) unchanging in their abilities and behaviour; and (5) emotionless.

**Table 2:** Summary of selected empirical dynamic capabilities research with supporting theoretical lenses

**Author(s)/year**	**Research focus**	**Theoretical lens**	**Research approach**
Altay *et al.* (2018)	To examine the effects of supply chain agility and supply chain resilience on performance under the moderating role of organizational culture	Organizational culture	Quantitative, survey of organizations involved in humanitarian operations
Arend (2014)	To examine the dynamic capabilities within entrepreneurial ventures and to investigate further the differences in how dynamic capabilities benefit firm performance	Entrepreneurship	Quantitative, survey of SMEs in the USA
Battisti and Deakins (2015)	To investigate the role of dynamic capabilities in a post‐disaster environment	Proactive posture and resource integration	Quantitative, survey of small firms in a post‐disaster environment
Brusset and Teller (2017)	To examine how resilience can be achieved by mapping the relationships between the practices, resources and processes over which a manager has control	Resource‐based view (RBV)	Quantitative, survey of supply chain managers
Cezarino *et al.* (2019)	To examine the factors that support the development of dynamic capabilities towards sustainable management	Systems thinking	Qualitative, single case study
Chen and Chang (2013)	To explore the influences of green dynamic capabilities and green transformational leadership on green product development performance, and investigate the mediation role of green creativity	Green creativity	Quantitative, survey of electronic industries in Taiwan
El Baz and Ruel (2021)	To investigate the role of supply chain risk management in mitigating the effects of disruption impacts on supply chain resilience and robustness in the context of COVID‐19 outbreak	Resource‐based view (RBV) and organizational information processing theory (OIP)	Quantitative, survey of French firms
Han and Li (2015)	To demonstrate the relationship between intellectual capital and innovative performance, and to specify the boundary conditions and mechanisms of the relationship from a knowledge‐based dynamic capability perspective	Knowledge‐based view (KBV)	Quantitative, survey of middle to senior managers in China
Karimi and Walter (2015)	To examine the role of dynamic capabilities in the performance of response to digital disruption	Disruptive innovation theory	Quantitative, survey of senior executive of newspaper companies
Karman and Savanevičienė (2021)	To develop and examine a model in which dynamic capabilities affect sustainable competitive advantage via sustainable practices and the mediating role of organizational ambidexterity	Sustainable competitive advantage and organizational ambidexterity	Quantitative, survey of organizations from the Baltic region
Lee, Kung and Li (2015)	To examine the development of dynamic capabilities in service multi‐units with different cultural distances through the routines of embedded social capital and knowledge archetype	Social capital	Quantitative, survey of MNCs in the Taiwanese service industry
Modgil, Singh and Hannibal (2021)	To examine how artificial intelligence is considered and employed by organizations to enhance supply chain resilience	Artificial intelligence (AI)	Qualitative, semi‐structured interviews

However, supply chain risk management (SCRM) is essentially human‐centric, where the success of any strategy relies heavily on individuals and/or teams to evaluate risk sources, to reduce the probability and consequences of their occurrence (Manuj and Mentzer, 2008). These ‘traditional’ models are not well equipped to explain the role of individuals, who are ultimately responsible for understanding and processing risks, in taking mitigative actions (Cantor, Blackhurst and Cortes, 2014). In this regard, the literature has highlighted the importance of studying the effect of human behaviour on supply chains’ and firms’ performances through behavioural research to address the gaps between the prediction of salient theories and actual practices (Sarafan, Squire and Brandon‐Jones, 2019; Tangpong, Hung and Li, 2014). Nonetheless, no prior research has looked closely into *how* risk perceptions as a behavioural aspect may facilitate dynamic capabilities within resilience.

To deal with limited information and substantive uncertainties, actors form subjective perceptions of risks that rely on a range of socio‐psychological processes and decision heuristics (Sitkin and Pablo, 1992). Mostly influenced by past experiences (Kull, Oke and Dooley, 2014) and organizational environments (Grudinschi, Sintonen and Hallikas, 2014; Smallman, 1996), risk perception has been regarded as a psychological factor that affects the efficacy of risk management and resilience strategies (DuHadway, Carnovale and Kannan, 2018), particularly during equivocal environments (Sarafan, Squire and Brandon‐Jones, 2019; Zsidisin, 2003; Zsidisin and Wagner, 2010).

Equivocality denotes the extent to which multiple meanings are linked with uncertain situations where information is limited and arises when ‘(i) derived meanings are subject to infinite revisions as events unfold and conflicting individual and social explanations are invoked, and (ii) relative superiority of a particular explanation remains ambiguous’ (Weick, 2001, p. 10). During these situations, heuristics influence risk perceptions as cognitive shortcuts by simplifying mental strategies for quick and efficient information processing (Newell and Simon, 1972; Visschers and Siegrist, 2008). These heuristics or ‘simple rules’ – such as Yahoo's rules for alliance formation (Rindova and Kotha, 2001) and Omni's rules for charter change (Galunic and Eisenhardt, 2001) – enable flexibility, yet coherent capture of problems and issues that guide decision‐making and actions.

Further research suggests that heuristic‐based risk evaluation is generally established on peculiar features such as previous knowledge and experiences (Maldonato and Dell'Orco, 2011). Bingham and Eisenhardt (2011) further highlight that firms can learn and utilize heuristics through organizational process experiences. In this sense, the relevance of organizational structures as the conditions that enable or constrain individual and collective interactions and communications (Felin *et al.*, 2012) becomes a salient component that affects SCR perceptions and decision‐making (DuHadway, Carnovale and Kannan, 2018; Kull, Oke and Dooley, 2014). Based on hierarchies, the literature has divided organizational structures into their most fundamental forms: authority‐based and consensus‐based (Nickerson and Zenger, 2004). These different structures will exhibit distinct communication flows and decision‐making processes which dictate the extent to which knowledge and information are transmitted (Felin *et al.*, 2012). When compared to authority‐based structures, the horizontal communication flows within consensus‐based structures increase the likelihood of knowledge integration (Arrow, 1974), which in turn promotes lower information costs, namely the ‘cost of collecting and processing information needed for output and investment decisions […]’ (Casson, 1999, p. 86).

The above discussion on previous literature highlights the importance of risk perceptions within dynamic capabilities and resilience decision‐making based on heuristics and organizational structures. However, organizational literature based on risk perceptions remains scant (DuHadway, Carnovale and Kannan, 2018; Kull, Oke and Dooley, 2014; Sarafan, Squire and Brandon‐Jones, 2019). Therefore, a behavioural lens of enactment theory that details the sensemaking process within managerial risk perception allows an investigation into how these processes may assist the phases and assembly of dynamic capabilities.

### Enactment theory

Enactment theory (Weick, 1995, 2001) has been regarded as a rich theoretical background to showcase the behavioural process of managerial risk decision‐making during equivocal environments (Ellis, Shockley and Henry, 2011; Olcott and Oliver, 2014; Sarafan, Squire and Brandon‐Jones, 2019). It proposes that experiences shaped by psychological and social processes determine how individuals and organizations ‘make sense’ of their environments (Kieran, MacMahon and MacCurtain, 2022; Mayson and Barrett, 2017; Weick, 1995, 2001). Sensemaking, being the foundation of enactment theory, is explained through its closed‐loop socio‐psychological process comprising enactment (i.e. actions and previous understandings that provide ‘raw’ materials for events), selection (i.e. attachment of meanings to actions by constructing plausible stories) and retention (i.e. storage of cause–effect relationships that inform future judgements) activities (Weick, 1969).

As a result, authors have emphasized psychological and social factors within organizational environments which affect managerial actions and decision‐making. For instance, using identity enactment theory, Thatcher and Zhu (2006) suggest that actors’ exposure to ‘psychologically strong situations’ within organizations (e.g. formal regulations enforcement and informal norms) acts as an external reference for their perceptions and actions. By combining institutional theory with enactment theory, Jensen, Kjærgaard and Svejvig (2009) investigate the implementation of information technology in hospitals and highlight how these systems shape actors’ identities and authorities which influence their actions. In the context of supply disruptions, Ellis, Shockley and Henry (2011) propose that decentralization and team diversity could reduce the level of equivocality, which in turn influences perceptions of supply risk. Similarly, Oliveira and Handfield (2017) suggest that open communication with suppliers can improve risk perception. However, despite growing acknowledgement of and interest in the use of enactment theory and sensemaking within organizational research, the literature is still in its infancy (Kieran, MacMahon and MacCurtain, 2022; Sarafan, Squire and Brandon‐Jones, 2019). Specifically, the understanding of how sensemaking can assist managerial risk perception and decision‐making within organizational resilience is absent.

## Theoretical background: Sensemaking in dynamic capabilities

As sensemaking ‘concerns the psychological and social processes through which individuals derive meaning from their experiences’ (Ellis, Shockley and Henry, 2011, p. 82), it fits naturally with dynamic capabilities, which require the process of acquiring knowledge resources and information in order to generate an understanding of uncertain environments prior to developing any responses (Eisenhardt and Martin, 2000; Kogut and Zander, 1992). Therefore, we draw similarities between the sensemaking process and dynamic capabilities, since both theories concern individuals’ and organizations’ ability to understand external environments to accordingly respond and adapt to the perceived environment (Teece, Pisano and Shuen, 1997; Weick, 1995).

**Figure 1:**
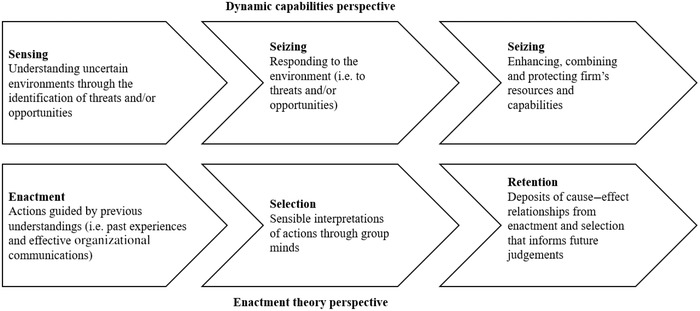
Theoretical linkage between dynamic capabilities and enactment theory

The execution of actions within the ‘enactment’ process, related to building close and effective communication and interconnectivity within and outside organizations, is essential so that actors can recognize different signals in equivocal environments (Oliveira and Handfield, 2017). This information facilitation can thus aid the understanding of uncertain environments and identification of risks during the phase of ‘sensing’. Commonalities across individuals’ cognitive maps as a result of effective communication can guide the ‘selection’ of sensible interpretations of actions (Weber and Glynn, 2006), which therefore can accommodate the adoption of appropriate measures to ‘seize’ the sensed risks. Lastly, the retention of cause–effect relationships associated with risks and responses can further inform the judgement of the reconfiguration of tangible and intangible assets during ‘transforming’. Together, the combination of dynamic capabilities and sensemaking provides us with a preliminary research model to study the resilience of organizations to SCRs during equivocal environments (Figure 1).

Drawing on this point, we argue that taking the behavioural perspective to further expand our understanding of organizational resilience to SCRs in equivocal environments is a valuable contribution to theory and practice (Ellis, Henry and Shockley, 2010; Ellis, Shockley and Henry, 2011; Sarafan, Squire and Brandon‐Jones, 2019), and further aims to address the limitations of prior studies identified by scholars, as indicated in the above sections.

## Methodology

### Research context

This qualitative study explores the underlying managerial sensemaking processes accounting for organizational resilience to SCRs in the UK food sector during the COVID‐19 pandemic. The rare global crisis of COVID‐19 has yielded a unique worldwide disruption context (Ruel *et al.*, 2021). The food sector is considered particularly valuable, as it displays resiliency through functionality and continuity assurance along its chains, despite experiencing major disruptions (OECD, 2020). The complications of SCRs in the food industry are significantly more profound compared to imperishable chains given their traits of seasonality, supply spikes and perishability (Behzadi *et al.*, 2018). This pandemic heightens these complications due to the lack of information regarding (i) the nature of the event, (ii) the causal relationship of the event and (iii) the availability of mitigation plans (Milliken, 1987), thereby displaying an environment that is equivocal.

The UK setting was selected for its unique traits, including self‐sufficiency concerns due to a heavy reliance on European imports and the remnants of Brexit, which could complicate and alleviate COVID‐19 impacts (Do *et al.*, 2021; Garnett, Doherty and Heron, 2020). Despite challenges, the UK's FSCs have been found to demonstrate a high degree of resiliency (Mitchell *et al.*, 2020).

The qualitative research design was chosen for its ability to generate a rich and coherent analysis of managers’ abilities and motivations, and the recognition of differences between consensus‐based and authority‐based organizational structures with reference to managerial behaviour and mental models (Eisenhardt, 1989; Miles, Huberman and Saldana, 2018). The sample aims to draw these variations in a process‐oriented manner and allows for the study of decision‐making with heterogeneous organizational structures and outcomes.

### Data collection

#### Sampling

To achieve an organizational resilience theory in equivocal environments and to elaborate the different conditions (i.e. consensus‐based and authority‐based organizational structures) under which such a theory operates, primary data is collected (Miles, Huberman and Saldana, 2018). In identifying our chosen firms, we follow Nickerson and Zenger (2004) by fundamentally differentiating organizational structures based on their hierarchies. An authority‐based hierarchy typically relies on centralized decision‐making activities and vertical communication flows (Arrow, 1974), whereas a consensus‐based hierarchy constitutes cross‐functional teams, which promotes horizontal communication channels among different functions (Hoopes and Postrel, 1999). With this rationale, the data collection began by contacting firms within the UK's food industry supply chain tiers which fall into our criteria of both consensus‐based and authority‐based structures through a variety of different professional networks. We then seek the appropriate respondents (i.e. directors and managers directly involved in the risk decision‐making processes before and during COVID‐19 periods) within the selected organizations, who further validated our categorization of the two organizational structures. This is conducted by firstly explaining the difference between the two structures at the beginning of each interview, and thereafter asking each interviewee to describe their organizational structure to further verify our categorization. Thus, by using theoretical sampling, interviewees were purposively sampled rather than randomly selected to enable comparability and variance of the relevant concepts (Eisenhardt and Graebner, 2007). This allowed adjustment and expansion of the sample throughout the data collection and analysis process (Corbin and Strauss, 2014).

#### Semi‐structured interviews

Between April and June 2020, 40 semi‐structured interviews (Table 3) were conducted, which varied in duration from 45 to 70 minutes. Such an interview sample is comparable to similar studies, adequately reflects the heterogeneity of manager views and represents the food industry's organizational resilience capabilities and managerial risk perception aspects during major global crises (Saunders and Townsend, 2016). Due to social distancing measures, all interviews were carried out via Internet‐based conference calls. Semi‐structured interviews following an interview protocol (see Table 4) allowed the structuring of discussions and facilitated the exploration of emerging themes (Charmaz, 2014). Notes were made during the interviews on issues raised, and the immediate thoughts of the researchers. Anonymity and confidentiality were assured, while informed consent was obtained via email.

**Table 3:** Interviewee profiles

**Interviewee**		**Organizational structure**	**Sector**	**Position**	**Years of experience**	**Interviewee**		**Organizational structure**	**Sector**	**Position**	**Years of experience**
1	A	Consensus‐based	Horticulture and packaging	Director of operations	22	21	U	Consensus‐based	Agriculture and processing	Director of supply chain and operations	31
2	B	Consensus‐based	Retail	Director of supply chain and operations	29	22	V	Consensus‐based	Retail	Director of operations	20
3	C	Authority‐based	Processing	Senior operations manager	20	23	W	Authority‐based	Processing	Director of logistics and supply chain	10
4	D	Consensus‐based	Produce packing	Director of logistics and operations	30	24	X	Authority‐based	Processing	Senior operations manager	16
5	E	Consensus‐based	Produce packing	Director of operations	30	25	Y	Consensus‐based	Processing	Director of supply chain and operations	17
6	F	Authority‐based	Processing	Director of operations	34	26	Z	Consensus‐based	Retail	Director of operations	23
7	G	Consensus‐based	Horticulture and packing	Director of supply chain	16	27	AA	Consensus‐based	Processing	Director of operations	14
8	H	Authority‐based	Processing	Director of operations	15	28	AB	Authority‐based	Horticulture and packing	Senior supply chain manager	13
9	I	Consensus‐based	Processing	Director of supply chain and logistics	26	29	AC	Authority‐based	Horticulture and packing	Supply chain and operations manager	22
10	J	Authority‐based	Processing	Director of operations	30	30	AD	Consensus‐based	Retail	Senior operations manager	13
11	K	Authority‐based	Processing	Senior operations manager	34	31	AE	Consensus‐based	Retail	Senior supply chain manager	21
12	L	Consensus‐based	Processing	Operations manager	22	32	AF	Consensus‐based	Agriculture	Senior supply chain manager	17
13	M	Authority‐based	Processing	Senior supply chain manager	31	33	AG	Authority‐based	Processing	Director of operations	20
14	N	Authority‐based	Processing	Director of supply chain and logistics	32	34	AH	Consensus‐based	Processing	Director of logistics and supply chain	23
15	O	Consensus‐based	Retail	Director of operations	23	35	AI	Consensus‐based	Processing	Director operations	33
16	P	Authority‐based	Processing	Senior operations manager	25	36	AJ	Consensus‐based	Retail	Senior operations manager	18
17	Q	Consensus‐based	Agriculture	Senior supply chain manager	20	37	AK	Consensus‐based	Horticulture and packing	Senior operations manager	16
18	R	Authority‐based	Horticulture and packing	Senior supply chain manager	21	38	AL	Authority‐based	Processing	Director of supply chain and logistics	19
19	S	Consensus‐based	Processing	Operations manager	10	39	AM	Consensus‐based	Retail	Senior supply chain manager	15
20	T	Consensus‐based	Processing	Director of operations	38	40	AN	Authority‐based	Retail	Senior supply chain manager	13

**Table 4:** Interview protocol

**Section**	**Questions**
Introduction	‐ Please provide a brief overview of your company and your responsibilities within the company
Prior to COVID‐19: Normal business operations	‐ Can you describe the organizational structure of the company? ‐ Prior to the COVID‐19 outbreak, how did your company with that structure normally identify and manage supply chain risks?
During COVID‐19: Sensing through enactment	‐ In general, what do you think of the impacts of the emerging COVID‐19 outbreak on your operations? ‐ Please provide the types of risks that your firms are currently facing. How did you identify these risks, for example, from reliable information sources or gut‐feeling or past experiences? ‐ To what extent does the existing organizational structure influence this risk identification stage?
During COVID‐19: Seizing through selection	‐ After these risks were identified, how did you evaluate and respond to them? Did these responses come from existing written procedures, or past experiences or any other sources, for example, observing actions of other firms? Please provide an example of a particular type of risk to elaborate this point. ‐ To what extent does the existing organizational structure influence your formulation of responding strategies? ‐ Are there any additional factors that strongly influence your success in formulating the response(s) during pandemic disruptions like COVID‐19?
Post COVID‐19: Assessment & transformation through retention	‐ In your view, how will this COVID‐19 pandemic affect your organization, risk management process and resilience level in the short and long term?

#### Additional data sources

The interview transcripts with actors are the main data source. They were triangulated with two other sources: (i) archival materials available online and those which were provided by the informants; and (ii) expert validation, for avoiding retrospective bias (Golden, 1992), to verify whether our impressions during the interview process were reflected in the secondary data sources. Specifically, archival materials provided background information on the companies, including their risk management rules and procedures. We had access to documents capturing the risk management procedures, which enabled a better understanding of the approaches prior to the COVID‐19 period. We contacted four experts from two consulting firms in the UK food sector through professional networks to conduct an expert validation step, including informal discussions through Internet‐based calls to assure the internal validity of the study's findings and conclusions (Golden, 1992).

### Data analysis

An abductive approach is a non‐linear and iterative process of collecting and analysing data to match theory with reality (Dubois and Gadde, 2002). We adopted this method to compare the formation of a new framework with extant literature‐based theory and new evidence from this study. Abductive inquiry is particularly suitable for pursuing theory development (i.e. refining existing theories, when the researchers have prior knowledge of suitable theoretical concepts but are open to new insights) (Miles, Huberman and Saldana, 2018; Van Echtelt *et al.*, 2008).

**Figure 2:**
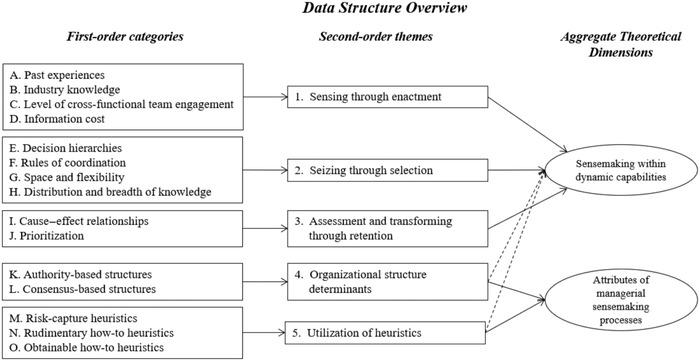
Data structure

NVivo 10, a computer‐assisted qualitative data analysis software, was used to organize, structure and code all collected data mentioned above. Data were first coded individually using an open‐coding method to allow the emergence of salient themes and capture the diversity and subjective nature of risks (Corbin and Strauss, 2014; Miles, Huberman and Saldana, 2018). A constant comparison technique by Corbin and Strauss (2014) was then adopted to travel back and forth between theory and data, to pursue a robust and comprehensive theoretical framework (Charmaz, 2014). The codes were repetitively re‐read alongside the original transcripts to isolate the relevant concepts and variables, and to discern differences and similarities among respondents. Codes were arranged in a hierarchical structure with 15 first‐order categories and five second‐order themes that were associated with two aggregated dimensions: sensemaking within dynamic capabilities and attributes of managerial sensemaking processes (Figure 2). We considered the higher‐level construct of organizational resilience as a summation of lower‐level managerial decision‐making, captured through enactment theory (Weick and Roberts, 1993). This permitted an exploration of the underlying managerial sensemaking cognitions that affect SCR decision‐making activities in the specific context of COVID‐19, ultimately accounting for organizational resilience.

### Research quality

We adopted measures in line with those proposed by Aguinis and Solarino (2019) and Gibbert, Ruigrok and Wicki (2008) to enhance the transparency and replicability of the study. Table 5 summarizes the measures taken to ensure methodological rigour.

**Table 5:** Methodological rigour

	**Procedures for rigour criteria in this study**		
**Rigour criteria**	**Design**	**Data collection**	**Data analysis**
Construct validity *(suitable measures for the concepts being studied)*	‐ Interview questions derived from previous research on resilience, dynamic capabilities and enactment theory	‐ Use multiple sources of data (interview data, workshop and secondary data) ‐ Maintain close dialogue with experts ‐ Pre‐test the interview protocol with academics and experts	‐ Triangulate data from multiple sources ‐ Use an abductive data coding process to enable novel themes to emerge ‐ Establish clear data coding and data analysis procedures ‐ Interview transcript validated by informants to avoid researcher bias
Internal validity *(causal relationships between variables and results)*	‐ Develop a framework based on well‐established resilience, dynamic capability and enactment theory literature	‐ Transcribe all interviews and send to respondents for checking ‐ Keep memos that focus on the perceptions and decision‐making process of informants	‐ Record alternative explanations ‐ Triangulate multiple theories for interpretation ‐ Travel back and forth between the data and literature to avoid researcher bias
External validity *(generalization of findings)*	‐ Select companies in multi‐echelon FSC while allowing for heterogeneity within different echelons	‐ Disclose sampling procedures ‐ Describe the research setting	‐ Pattern matching for analytical generalization (matching the resulting patterns of dynamic capabilities and sensemaking to those discussed in the literature)
Reliability *(replicability of the research design and result)*	‐ Develop a consistent and clear interview protocol ‐ Construct a written data collection and data analysis procedure	‐ Build a database to establish a chain of evidence (including transcripts, memos, secondary materials)	‐ Use NVivo 10 for data analysis and keep a record of the coding process ‐ Discuss interim results between researchers

## Findings and discussion

**Figure 3:**
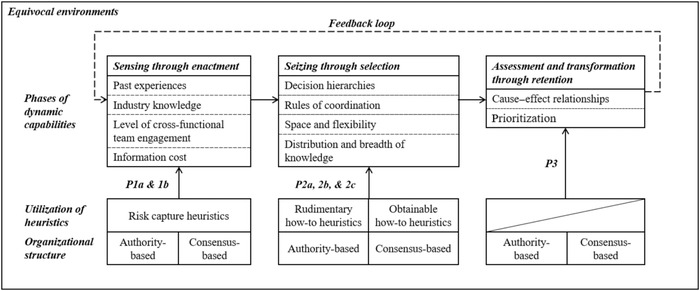
A framework of organizational resilience to supply chain risks during equivocal environments – sensemaking within dynamic capabilities

We structure our findings along the stages of dynamic capabilities during equivocal environments that were assisted by the phases of managerial sensemaking processes: sensing through enactment, seizing through selection, assessment and transformation through retention (see Table 6). We further explain the different heuristics utilized during phases of sensing and seizing that were contingent upon different organizational structures (see Table 7). This section discusses the identified patterns against the theoretical background at the outset and develops propositions aimed at condensing the main findings to an empirically grounded model of organizational resilience to SCRs during equivocal environments (see Figure 3).

**Table 6:** Themes, categories and representative data

**Second‐order themes**	**First‐order categories**	**Representative quotes**
Sensing through enactment	Past experiences	‘… I think I've been here [in the food industry] long enough to kind of know what are the risks that we will face. You know, there are other recent situations or events that have had as much of an impact on business … the impact on commodities, Brexit, trade deals and whatnot. The Greta Thunberg effect, plastics […] So COVID‐19 is just one of many experiences that have had an impact’ (Interview AD) ‘[we] identified the fallback restructure of the shift system to run with 50% of staff in the reduced demand period, covering with temporary staff if needed. We've had similar situations in the past where we had to do almost the same thing. But yes, the scale is totally different in this case’ (Interview W)
	Industry knowledge	*Authority‐based structures*: ‘The business always had a pandemic identified as a business risk but more from an internal operational perspective rather than the impacts on customers and customer demands. What COVID‐19 has taught us is the breadth and depth of the impact of a pandemic, like loss of customers, loss of demand overnight, etc.’ (Interview H) *Consensus‐based structures*: ‘The global nature of COVID‐19 has trumped usual commercial sensitivity across the sector and led to truly open communication throughout the supply chain. And at least for us, this also helps, in terms of information that we get from our partners’ (Interview AK)
	Level of cross‐functional team engagement	*Authority‐based structures*: ‘There is limited internal evaluation of risk. What there is, is predominantly from weekly and monthly financial figures with no formal structure to assessment and measurement of risk categories per se […] So I would say we struggle there. And for COVID we are getting some consultants in to help us with this’ (Interview R) *Consensus‐based structures*: ‘[we] redeploy[ed] resources from “closed” channels to those that are “open”. Our supplier portfolio is quite huge, you see. We did plan to reduce this, because we thought it's the best way to reduce cost, by streamlining our supplies, but thankfully we didn't. It's a very good idea in “normal” situations, which I did long back during the financial crisis, didn't work well obviously. Thankfully, we noticed it and it kind of saved us. Specially in the early days of lockdown’ (Interview AK)
	Information costs	‘Many of the outcomes of COVID‐19 were completely unforeseen and not planned for. Our IT infrastructure is a simple example. We were not equipped for online conferences calls. We prefer to do it the “old‐fashioned” way. So, it was a big hassle for us to try and scale this up. Manpower was difficult and don't even start with the cost’ (Interview AG) ‘There is limited internal evaluation of risk. What there is, is predominantly from weekly and monthly financial figures with no formal structure to assessment and measurement of risk categories per se […] So I would say we struggle there. And for COVID we are getting some consultants in to help us with this’ (Interview R)
Seizing through selection	Decision hierarchies	*Authority‐based structures*: ‘In my opinion, our system is definitely not optimal yet. You know, you can only see this when you're faced with situations like COVID. We leave everything to subject matter experts when it comes to risks. And I think we should be more open to have, you know, a more open discussions with our employees. We really negate the use of teams here. More brains are better when we're trying to resolve things at this scale’ (Interview W) ‘When you leave things to be resolved by only senior leaders, it kind of present[s] how our organization lacks transparency. I mean of course, in other parts we are quite transparent I would say. But, not for risks. Especially now during COVID’ (Interview H) *Consensus‐based structures*: ‘We realized how important it is to have this kind of culture or mindset if you like. We not only have clear communication, but also discuss issues across teams which I think is very valuable during times like this, especially you are talking about risks right. To see other perspectives and then see how we can use it together’ (Interview U)
	Rules of coordination	*Authority‐based structures*: ‘Our system is not well integrated enough to take account what everyone is doing at the moment. So, it's like we have to just make decisions to resolve issues at best we can alone […] what we hold on to is simply to increase flexibility and maybe just to simplify our current operations in each department, whatever that is’ (Interview N) *Consensus‐based structures*: ‘[we have a] business risk committee at parent (multi‐national) group level which maintains a comprehensive risk register includes financial risks and some other functional risks. The business also has a crisis management (business continuity) plan at both group and operating business levels. Coincidentally, shortly before COVID‐19 struck, one Division had completed crisis management retraining across the whole business, including remote working and movement of operations to co‐packers’ (Interview AJ)
	Space and flexibility	*Authority‐based structures*: ‘I mean, in a sense, we can be more flexible right. To do what we think it's best. We can try and see, for example to just focus on finance. More acute cash management […] changes to terms where possible with customers and suppliers, removal of non‐essential spending and CAPEX. And then see if it works or not’ (Interview M) *Consensus‐based structures*: N/A
	Distribution and breadth of knowledge	*Authority‐based structures*: ‘Again, it's the same thing as what I've said before. It's sad to say that we have to admit that we're lacking in that part. Not all of our employees have the same information as others, higher up. You won't know the impact of this until you're hit with a situation like COVID. We do aim to revamp this’ (Interview AB) *Consensus‐based structures*: ‘Ensuring clear communication, keeping a global team focused when some are furloughed others working and feeling at [personal] risk’, achieved through ‘regular team conference calls and monitoring of staff morale by [the] HR team’ and ‘Head Office having a greater focus on the [COVID‐19] issue in each region’ (Interview O)
Assessment and transformation through the retention	Cause–effect relationships	‘[It is] too early to say definitely what the long‐term changes will be, but a better understanding of supply chain resilience will feature, along with improved communication’ (Interview F) ‘No change so far. The organization is agile enough to quickly adapt and respond to changing operating climate. The biggest increased risks are the risk to people/welfare and possible supply chain disruption which then requires much more micro attention from the procurement team’ (Interview Q) ‘We see no change that we envisage, at least not for now. Everything is just still too uncertain, blurry if you like, to know what long‐term changes we want to make. Especially if you ask about permanent changes in our processes, we just don't know yet’ (Interview AL) ‘The pandemic risk assessment is being completely re‐written. The existing assessment was very “top line” reflecting a need to address issues around people, products, plant, and a communications plan. The new plan will have much greater depth and detail, informed by the experience [of] COVID‐19’ (Interview L)
	Prioritization	‘The company tries to simplify all tasks as much as possible and has started looking for technologies to either reduce dependence on repetitive labour‐intensive tasks or support workers being more effective’ (Interview AN) ‘Each scenario [herein refers to economic condition scenarios] has a set of response functions built in which will trigger switching between models as necessary’ (Interview P) ‘[we] initiated an early planning with agency labour providers, and guaranteed hours for seasonal agency workers to ensure availability of labour’ (Interview AA) ‘Back‐up plans created for Logistics and Operations activities redeploying non‐operational staff and alternative external contractors’ (Interview AC) ‘In late February, a company COVID‐19 risk committee was formed to manage policy review, changes and supply chain considerations. The company reports weekly to customers on COVID‐19‐related risk information in its own operations and supply chain’ (Interview D)
Organizational structure determinants	Authority‐based structures	‘Operational risks have been identified by functional and subject matter experts for each key business area. These risks are assessed quarterly at a Business Risk meeting attended by senior leaders’ (Interview J) ‘There is limited internal evaluation of risk. What there is, is predominantly from weekly and monthly financial figures with no formal structure to assessment and measurement of risk categories per se, and tends to be more ad‐hoc and reactive with little consideration of risks which are not directly commercial. So I would say we struggle there. And for COVID we are getting some consultants in to help us with this’ (Interview R) ‘Combination of traditional known risks (e.g. reputational damage, food safety incidents) and horizon scanning through media channels, contacts, suppliers and business organizations. Yeah we do meetings from time to time to see what other departments are doing, but honestly we don't really, our system is not well integrated enough to take account what everyone is doing at the moment. So it's like we have to just make decisions to resolve issues at best we can alone. The most important thing is not to minimize profit loss. What we hold on to is simply to increase flexibility and maybe just to simplify our current operations in each department, whatever that is. […] also for future planning and general business designs. You never know when this might happen again or how long it will last’ (Interview N)
	Consensus‐based structures	‘Ensuring clear communication, keeping a global team focused when some are furloughed others working and feeling at [personal] risk’, achieved through ‘regular team conference calls and monitoring of staff morale by [the] HR team’ and ‘Head Office having a greater focus on the [COVID‐19] issue in each region’ (Interview O) ‘[we have a] business risk committee at parent (multi‐national) group level which maintains a comprehensive risk register includ[ing] financial risks and some other functional risks. The business also has a crisis management (business continuity) plan at both group and operating business levels. Coincidentally, shortly before COVID‐19 struck, one Division had completed crisis management retraining across the whole business, including remote working and movement of operations to co‐packers’ (Interview AJ) ‘The outbreak has been managed using a Crisis team with verbal reports from function/subject matter experts from across the business. Past crisis management situations have typically only affected an individual site or a small number of operational functions, with many areas of the businesses unaffected and able to continue normal operations. The high transmission rate of this outbreak (unlike previous viral epidemics) has tested the resilience of business continuity planning across all departments and sites. So, in this case we really emphasize information transmission and also of course more group meetings’ (Interview A)
Utilization of heuristics	Risk‐capture heuristics	‘… I think I've been here [in the food industry] long enough to kind of know what are the risks that we will face. You know, there are other recent situations or events that have had as much of an impact on business … the impact on commodities, Brexit, trade deals and whatnot. The Greta Thunberg effect, plastics […] So COVID‐19 is just one of many experiences that have had an impact’ (Interview AD) ‘[we] identified the fallback restructure of the shift system to run with 50% of staff in the reduced demand period, covering with temporary staff if needed. We've had similar situations in the past where we had to do almost the same thing. But yes, the scale is totally different in this case’ (Interview W) ‘Financially, we are having internal issues because we're struggling to maintain “business as usual”. And of course, one of the risks for this would be trying to maintain our relationships with our key customers. If we don't deliver our performances as usual, this will be an issue. But I think since everyone is also impacted by COVID, it's not just us who's facing this risk, but our customers are also experiencing the same thing’ (Interview J) ‘… consequently of course we have risks in our operations. Simple things like, because of the risk of high or low stocks in our inventory and manufacturing cycle we will have problems in delivery lead times. To be honest this is not new. I would even say this is also somewhat like running the risks in our usual day to day operations, but the difference is that the scale is so much bigger’ (Interview X)
	Rudimentary how‐to heuristics	‘The company tries to simplify all tasks as much as possible and has started looking for technologies to either reduce dependence on repetitive labour‐intensive tasks or support workers being more effective’ (Interview AN) ‘I mean, in a sense, we can be more flexible right. To do what we think it's best. We can try and see, for example to just focus on finance. More acute cash management […] changes to terms where possible with customers and suppliers, removal of non‐essential spending and CAPEX. And then see if it works or not’ (Interview M) ‘Back‐up plans created for Logistics and Operations activities redeploying non‐operational staff and alternative external contractors’ (Interview AC) ‘Our system is not well integrated enough to take account what everyone is doing at the moment. So, it's like we have to just make decisions to resolve issues at best we can alone […] what we hold on to is simply to increase flexibility and maybe just to simplify our current operations in each department, whatever that is’ (Interview N)
	Obtainable how‐to heuristics	‘[we] redeploy[ed] resources from “closed” channels to those that are “open”. Our supplier portfolio is quite huge, you see. We did plan to reduce this, because we thought it's the best way to reduce cost, by streamlining our supplies, but thankfully we didn't. It's a very good idea in “normal” situations, which I did long back during the financial crisis, didn't work well obviously. Thankfully, we noticed it and it kind of saved us. Specially in the early days of lockdown’ (Interview AK) ‘[we have a] business risk committee at parent (multi‐national) group level which maintains a comprehensive risk register includ[ing] financial risks and some other functional risks. The business also has a crisis management (business continuity) plan at both group and operating business levels. Coincidentally, shortly before COVID‐19 struck, one Division had completed crisis management retraining across the whole business, including remote working and movement of operations to co‐packers’ (Interview AJ) ‘In late February, a company COVID‐19 risk committee was formed to manage policy review, changes and supply chain considerations. The company reports weekly to customers on COVID‐19‐related risk information in its own operations and supply chain. Too early to say definitively what the longer‐term changes will be, but a better understanding of supply chain resilience will feature, along with improved communication’ (Interview D)

**Table 7:** Characteristics of heuristics in sensemaking and dynamic capabilities during equivocal environments

**Dynamic capability phase**	**Sensing through enactment**		**Seizing through selection**	
**Organizational structure**	**Authority‐based structures**	**Consensus‐based structures**	**Authority‐based structures**	**Consensus‐based structures**
**Heuristics type**	**Risk‐capture heuristics**		**Rudimentary how‐to heuristics**	**Obtainable how‐to heuristics**
Definition	Rules of thumb that guide the identification of risks in the phase of sensing through enactment of actions – derived through past experiences and industrial knowledge – that are related to building close and effective communication and interconnectivity within and outside the organization.		Basic how‐to rules of thumb detailing the actions to resolve or mitigate the identified risks derived through the space and flexibility created from limited horizontal informational and knowledge sharing.	Communal how‐to rules of thumb detailing actions to resolve or to mitigate identified risks derived through the rules of coordination from strong horizontal information and knowledge sharing.
Examples	Association of risks to similar previous experiences (e.g. Brexit, trade deals, Greta Thunberg effects) Association of risks with the information gathered from industry knowledge (e.g. market and partner engagements, external consultants, trade bodies)		More acute cash management Task simplifications Back‐up plans for logistics and operations Maintain constant liaison with shipping lines Inform customers of delays	Business risks committee Crises management plans Economic condition scenarios Redeploy resources from closed channels to open channels Ensure clear communication
Reasons for importance	Risk‐capture heuristics assist managers to identify risks when information is limited by enacting actions related to effective communications derived through past experiences and industrial knowledge. Without risk‐capture heuristics, actors may struggle to identify risks during equivocal environments.		Rudimentary how‐to heuristics assist managers to identify and organize actions derived through space and flexibility in resolving or mitigating the identified risks. Without rudimentary how‐to heuristics, actors may increase time for decision‐making due to the lack of information and misunderstanding of risks.	Obtainable how‐to heuristics assist managers to identify and organize actions derived communally in resolving or mitigating identified risks. Without obtainable how‐to heuristics, actors may pursue simpler actions through the utilization of rudimentary how‐to heuristics.

### Sensing through enactment: Identifying SCRs during COVID‐19

This study shows that pandemic interruptions to business operations affected the flow of information. The absolute availability of some information, its timeliness and reliability, served to further complicate operational continuity. In many cases, actors were simply unable to provide reliable information given the cumulative uncertainties along value chains.

According to behavioural research, when information is limited, heuristics are often deployed as cognitive shortcuts to capture problems or opportunities that make decision‐making more effective (Eisenhardt and Sull, 2001). For example, heuristics can be used as boundary rules to select which opportunities to pursue, leave or ignore (Bingham and Eisenhardt, 2011). Heuristics are essential in unpredictable environments (Davis, Eisenhardt and Bingham, 2009), particularly when perceiving risks. Similarly, our findings show that interviewees utilize *risk‐capture heuristics* consisting of boundary rules to identify SCRs associated with COVID‐19 through past experiences. With an average of more than 20 years on‐the‐job practice in the food industry, our research identified managerial experience as an important predictor in deploying perceptions (John and Björkman, 2015; Kooij and Boon, 2018), and in our case ‘horizon scanning’, crucial to notice and bracket limited available data that informs the identification of risks:

… I think I've been here [in the food industry] long enough to kind of know what are the risks that we will face. You know, there are other recent situations or events that have had as much of an impact on business … the impact on commodities, Brexit, trade deals and what not. The Greta Thunberg effect, plastics […] So COVID‐19 is just one of many experiences that have had an impact. (Interview AD)

This experience prompted the utilization of the risk‐capture heuristics: identification of risks through repeating actions from similar past situations such as Brexit and trade deals. The interviewees used these heuristics for the identification of risks during an equivocal environment.

We further extend this by highlighting the elements that inform these heuristics, associated with different organizational structures in place. Following Felin *et al.* (2012), we define organizational structures as conditions that enable or constrain individual and collective interactions within an organization, which in turn will affect the dissemination of knowledge and information.

When reducing equivocality in identifying risks, the interviewees state that the consensus‐based structures seem to have an advantage, having enjoyed the functionality of strong cross‐functional team culture. This is further strengthened through their frequent partner and market engagements, which have proved helpful to identify and discuss SCRs at hand. Therefore, these activities display a frequent enactment of previous and current actions (Weick, 2001):

The global nature of COVID‐19 has trumped usual commercial sensitivity across the sector and led to truly open communication throughout the supply chain. And at least for us, this also helps, in terms of information that we get from our partners. (Interview AK)

Furthermore, these companies have always concentrated on factors that might affect the profit and turnover of the organizations through specific risk assessment systems (e.g. threat assessment, critical control points and hazard analysis – systems which are an industry‐wide management process). This resonates with prior literature, which underlines the importance of digital technologies to ensure organizational and supply chain visibility in securing business continuity during extreme disruption (i.e. COVID‐19) (Papadopoulos, Baltas and Balta, 2020; Yang *et al.*, 2021), such as organizational information and decision support systems, which enable coping strategies (Ellis, Shockley and Henry, 2011; Zsidisin *et al.*, 2004).

Conversely, authority‐based structures struggle at gathering industrial knowledge and information to reduce equivocality during COVID‐19. The majority of authority‐based structures seemed to enact actions by investing heavily in acquiring additional assistance from external agents (i.e. insurance brokers, external consultants, trade bodies) to provide information and additional support during the outbreak. Moreover, by having limited organizational controls in terms of cross‐functional team engagement culture, these organizations were further confronted with difficulties in enabling effective communication:

There is limited internal evaluation of risk. What there is, is predominantly from weekly and monthly financial figures with no formal structure to assessment and measurement of risk categories per se […] So I would say we struggle there. And for COVID we are getting some consultants in to help us with this. (Interview R)

Overall, both structures can ‘sense’ or identify COVID‐19‐related risks through the enactment of building or having established effective communications, assisted by the utilization of risk‐capture heuristics. Furthermore, to reduce environmental equivocality, authority‐based structures invest more (enact more actions) to receive informational support, compared to the already existing horizontal communication channels within consensus‐based structures, resulting in higher information costs as analysed above.

*P1a*: During equivocal environments, organizations (individuals) can sense or guide their recognition of SCRs through the utilization of risk‐capture heuristics and by enacting actions related to effective communications that are contingent upon the level of cross‐functional teams’ engagement and industrial knowledge.
*P1b*: Due to vertical communication channels, authority‐based structures are more likely to expect higher information costs as compared to consensus‐based structures that already exhibit existing horizontal communication channels.

### Seizing through selection

The interviews show differences in the heuristics utilized during the seizing phase of the two types of organizational structures, as they seldom have equal levels of information acquisition and dissemination abilities. Instead, there is a trade‐off between these abilities and the availability of space and flexibility created that are associated with specific developed heuristics. This ability is derived through decision hierarchies and rules of coordination based on the organizational structure in place. These structures dictate the level of organizational space and flexibility, which in turn affect the distribution and breadth of information and knowledge, thereby extending the basic assumption that organizational structure reduces environmental equivocality by facilitating sensible information and group minds (Ellis, Shockley and Henry, 2011; Weick, 2001).

Our interviewees admit that although the authority‐based structures offer basic systems (e.g. risk registers) to support their decision‐making, their risk management decision hierarchies and processes remain siloed with limited internal evaluations, lacking standardization and rules of coordination. Structure in place only allows ‘senior leaders’ or ‘subject matter experts’ to assess appropriate actions towards the identified risks. They also show that this process does not have a ‘clear pathway’ (Interview AG), ‘lacks transparency’ (Interview H) and further ‘negates the use of teams’ (Interview W). Management scholars have argued that authority‐based structures do not promote the horizontal communication channels needed to support such knowledge sharing amongst peers (Nickerson and Zenger, 2004). Consequently, in turn they have limited distribution and breadth of knowledge.

However, in our case the limited space and flexibility created through the issues above induce actors to resort to simple rules, based upon individuals’ judgement as *rudimentary how‐to heuristics* in guiding them to select and execute actions to eliminate plausible options:

Our system is not well integrated enough to take account what everyone is doing at the moment. So, it's like we have to just make decisions to resolve issues as best we can alone […] what we hold on to is simply to increase flexibility and maybe just to simplify our current operations in each department, whatever that is. (Interview N)

Other respondents have also mentioned the utilization of these simple rules, which focus on financial management through initiating warehouse outsourcing and local material sourcing, as ‘additional short‐term stocks of key high‐demand items could be bought from local suppliers’ (Interview P). Interviewee M responds typically:

More acute cash management […] changes to terms where possible with customers and suppliers, removal of non‐essential spending and CAPEX.

Moreover, by relying on these heuristics, the managers’ courses of action selection within authority‐based structures allow them to select options for resolving the SCRs, which have ‘worked so far’ in resolving risks (Interview C). Our data showcased that although constrained by lack of horizontal communication amongst peers, these rudimentary how‐to heuristics can surprisingly be as accurate or even outperform analytically complicated and information‐intensive approaches considering that information, knowledge and time are available (Eisenhardt and Sull, 2001).

Conversely, the interviewees emphasize that the consensus‐based structures can acquire diverse knowledge sets by promoting peer information‐sharing. A consensus‐based hierarchy involves having individuals collectively agree on a search path and create a commonly shared language that integrates their specialized knowledge, via existing horizontal communication channels (Arrow, 1974). This shared identity facilitates a comprehensive group mindset (Weick, 2001), which enables rules of coordination to influence search and learning directions (Kogut and Zander, 1996) and reduces information costs. Typically comprised of cross‐functional teams, our respondents exhibit business continuity plans of crisis management within the group and operating business levels. This also involves strict procedures and controlled events on ‘what to do next’ when faced by certain SCRs:

[we have a] business risk committee at parent (multi‐national) group level which maintains a comprehensive risk register includ[ing] financial risks and some other functional risks. The business also has a crisis management (business continuity) plan at both group and operating business levels. Coincidentally, shortly before COVID‐19 struck, one Division had completed crisis management retraining across the whole business, including remote working and movement of operations to co‐packers. (Interview AJ)

The involvement of our respondents in cross‐functional teams challenges the actors’ bounded rationality by exposing them to new perspectives and ways of solving identified SCRs. Including the selection process of seizing identified SCRs, this structure allows sensible interpretations of actions through group minds (Ellis, Shockley and Henry, 2011; Weick, 2001), permitting actors to utilize obtainable how‐to heuristics:

We realized how important it is to have this kind of culture or mindset if you like. We not only have clear communication, but also discuss issues across teams which I think is very valuable during times like this, especially you are talking about risks right. To see other perspectives and then see how we can use it together. (Interview U)

Furthermore, the ‘availability’ of heuristics is not only derived as an individual experience, but a communal one. As the process of sensemaking involves a selection of cues based on familiar situations (Weick, 1995, 2001), by being exposed to multiple perspectives through meetings and peer discussions, actors can select cues on the basis of obtainable how‐to heuristics within the organization. The dispersed power and decision‐making apparatus of consensus‐based hierarchies creates resources (in our case information) more readily available to support managers in their decision‐making (Felin and Zenger, 2014).

Finally, we note that organizational structures, regardless of type, allow the production of different heuristics for managers when selecting appropriate actions for identified SCRs during the pandemic.

*P2a*: Regardless of the type, both authority‐based and consensus‐based organizational structures serve as a platform that allows the production of different heuristics that can be used to seize the SCRs through actions/responses and to further provide meaning, a contextually rational explanation, of the chosen actions/responses.
*P2b*: The space and flexibility created through the limited horizontal information and knowledge sharing of authority‐based structures enable the production and utilization of rudimentary how‐to heuristics, simple basic ‘how‐to’ rules that guide actors in selecting actions/responses to SCRs.
*P2c*: The established rules of coordination made through stronger horizontal information and knowledge sharing within consensus‐based structures enable the production and utilization of obtainable how‐to heuristics, the communally available ‘how‐to’ rules that guide actors in selecting actions/responses to the SCRs.

### Assessment and transforming: A process of retention

Our results extend prior resilience research that hinges on dynamic capabilities (e.g. Brusset and Teller, 2017; Eltantawy, 2016; van Essen *et al.*, 2015) by suggesting that both types of organizational structures can achieve resilience during equivocal environments, despite mixed findings within this section. Some organizations have begun to change their organization's risk management strategies accordingly, and some are still in the process of ‘assessment’. Considering our research question, our findings suggest that companies still being in the assessment stage before changing their routines does not necessarily mean that resilience cannot be achieved.

In relation to the sensemaking process, this assessment period before transformation depicts the retention process of objectifying the ‘plausible story’ or courses of action that have been deployed in the selection process (Weick, 1995, 2001). This is in line with prior studies that underline the importance of reducing equivocality through acquiring and developing information regarding cause–effect relationships (i.e. mitigative actions and their consequences) (Ellis, Henry and Shockley, 2010; Ellis, Shockley and Henry, 2011; Weick, 2001). Due to the nature of this study, by collecting data during the height of the pandemic, our respondents still have limitations regarding understanding the outcome of their actions (i.e. the cause–effect relationships).

Companies that have transformed their organization processes are taking a more holistic perspective of future potential risks, and how existing resources can be better applied to repeated systemic issues via prioritization, to decide which capabilities should be developed. This allows firms to manage their limited resources in developing dynamic capabilities (Zsidisin *et al.*, 2004):

In late February, a company COVID‐19 risk committee was formed to manage policy review, changes, and supply chain considerations. The company reports weekly to customers on COVID‐19‐related risk information in its own operations and supply chain. (Interview D)

Whilst others are also completely re‐writing their pandemic risk assessment tools, most existing transformations are very ‘top‐line’, reflecting a need to address issues around people, products, plants and communication plans. Other transformations also conducted by companies include more operational matters, such as individual training and development, management awareness training and having a ‘correct IT infrastructure’ capable of supporting a flexible workforce. Additionally, COVID‐19 highlights how quickly fear of the unknown can develop, putting businesses at significant risk, so organizations should ‘expect the unexpected’ (Interview B).

Some respondents also revealed that the transformations were not significant due to their successful responses to the crisis. For example, due to their Brexit preparation, risks of financial disturbance were reasonably balanced, as their budget‐setting had been conservative for some time (Interview AI). Others explained that by sticking closely to the government's advice and acting early and decisively, they were already more than three weeks ahead of most companies in their sector. Although these responses are labelled ‘conservative methods’, they imply that the organization has depicted resiliency by being able to respond successfully to the crisis.

However, some of the respondents raised awareness and concerns of the cost implications when incorporating risk management strategies for something that ‘only happens once in a lifetime’ (Interview P). In longer timeframes, some firms identified a need to regain control of direct labour sourcing and protect their workers, to avoid losing knowledge and experience. Although the COVID‐19 crisis is still ongoing, the response for organizational transformation of maintenance regarding continued renewal practices remains rather uncertain, due to their evaluation process. Our respondents’ responses are generally concerned with dealing with the present problems. And that:

[It is] too early to say definitely what the long‐term changes will be, but a better understanding of supply chain resilience will feature, along with improved communication. (Interview F)

*P3*: In equivocal environments, both authority‐based and consensus‐based organizations employ an assessment stage that depicts the cause–effect relationships of the committed action performed to the SCRs as a process of retention prior to the transformation that will further constrain and or influence the interpretation and selection of future actions and transformations.

## Contributions and implications

This paper sets out to develop a novel framework that explains how organizational resilience to SCRs can be achieved during equivocal environments caused by extreme uncertainties, focusing on investigating the role of managerial risk perceptions in assisting the assembly of dynamic capabilities. The results contribute to the current literature on resilience and sensemaking in several ways (see Table 8). First, by following the procedure for developing a construct definition based on prior literature (Ambulkar, Blackhurst and Grawe, 2015; Gilliam and Voss, 2013; Raetze *et al.*, 2021), we extract and compare extant definitions of resilience. By expanding resilience based on two different capability approaches (i.e. adaptation and anticipation) during equivocal environments, we contribute to current resilience research which suggests that organizational success and growth in crises can only be achieved by using different approaches in combination (Alikhani, Torabi and Altay, 2021; Conz and Magnani, 2020; Duchek, 2020; Williams *et al.*, 2017).

**Table 8:** Contributions, supporting studies and selective empirical evidence

		**Scope**			
**Theoretical contribution**	**Details**	**Sensemaking**	**Resilience and dynamic capabilities**	**Supporting studies**	**Selective empirical evidence**
Resilience definition during equivocal environments based on a capability approach	We extract and compare extant definitions of resilience that are based on dynamic capabilities within two different capability approaches (i.e. adaptation and anticipation) in order to provide a definition of resilience during equivocal environments	–	X	Refer to Table 1	N/A
A nuanced understanding of the construction of dynamic capabilities to achieve resilience during equivocal environments, with the utilization of the behavioural perspective of sensemaking	Sensemaking assists the construction of dynamic capabilities in equivocal environments via sensing through enactment, seizing through selection, and assessment and transformation through retention	–	X	Duchek (2020) Sarafan, Squire and Brandon‐Jones (2019) Eisenhardt and Martin (2000) Kogut and Zander (1996)	See columns below for detailed evidence of each of the phases
Production and utilization of heuristics within different types of organizational structures (authority‐based and consensus‐based)	Utilization of risk‐capture heuristics consisting of boundary rules in identifying risks associated through past experiences. The enactment of actions related to building close and effective communication and interconnectivity. Albeit authority‐based structures are confronted by higher information costs as compared to consensus‐based structures.	X	X	Eisenhardt and Sull (2001) Bingham and Eisenhardt (2011) Davis, Eisenhardt and Bingham (2009) Zsidisin *et al.* (2004) Ellis, Shockley and Henry (2011) Cantor, Blackhurst and Cortes (2014) Zsidisin and Wagner (2010)	‘… I think I've been here [in the food industry] long enough to kind of know what are the risks that we will face. You know, there are other recent situations or events that have had as much of an impact on business … the impact on commodities, Brexit, trade deals and whatnot. The Greta Thunberg effect, plastics […] So COVID‐19 is just one of many experiences that have had an impact’ (Interview AD) ‘The global nature of COVID‐19 has trumped usual commercial sensitivity across the sector and led to truly open communication throughout the supply chain. And at least for us, this also helps, in terms of information that we get from our partners’ (Interview AK) ‘There is limited internal evaluation of risk. What there is, is predominantly from weekly and monthly financial figures with no formal structure to assessment and measurement of risk categories per se […] So I would say we struggle there. And for COVID we are getting some consultants in to help us with this’ (Interview R)
	Utilization of how‐to heuristics during the seizing through selection phase that are contingent upon different organizational structures. Authority‐based structures can produce and use rudimentary how‐to heuristics through the space and flexibility created with limited horizontal communication flow. However, the exposure to group minds within the consensus‐based structures leads to the production and utilization of obtainable how‐to heuristics.	X	X	Weick (2001) Ellis, Shockley and Henry (2011) Nickerson and Zenger (2004) Zsidisin and Wagner (2010)	‘We realized how important it is to have this kind of culture or mindset if you like. We not only have clear communication, but also discuss issues across teams which I think is very valuable during times like this, especially you are talking about risks right. To see other perspectives and then see how we can use it together’ (Interview U) ‘[we have a] business risk committee at parent (multi‐national) group level which maintains a comprehensive risk register includ[ing] financial risks and some other functional risks. The business also has a crisis management (business continuity) plan at both group and operating business levels. Coincidentally, shortly before COVID‐19 struck, one Division had completed crisis management retraining across the whole business, including remote working and movement of operations to co‐packers’ (Interview AJ) ‘Our system is not well integrated enough to take account what everyone is doing at the moment. So, it's like we have to just make decisions to resolve issues at best we can alone […] what we hold on to is simply to increase flexibility and maybe just to simplify our current operations in each department, whatever that is’ (Interview N) ‘I mean, in a sense, we can be more flexible right. To do what we think it's best. We can try and see, for example to just focus on finance. More acute cash management […] changes to terms where possible with customers and suppliers, removal of non‐essential spending and CAPEX. And then see if it works or not’ (Interview M)
Assessment as an additional step in dynamic capabilities during equivocal environments	In relation to the sensemaking process this assessment phase depicts a ‘retention process’ of objectifying plausible stories or courses of actions that has been deployed in the seizing process. Due to the nature of our study, by collecting data during the height of the pandemic, respondents still have limitations in terms of understanding the outcomes of their actions (i.e. cause–effect relationships)	X	X	Weick (2001) Weick (1995) Ellis, Henry and Shockley (2010) van Essen *et al.* (2015) Eltantawy (2016)	‘We see no change that we envisage, at least not for now. Everything is just still too uncertain, blurry if you like, to know what long‐term changes we want to make. Especially if you ask about permanent changes in our processes, we just don't know yet’ (Interview AL) ‘[It is] too early to say definitely what the long‐term changes will be, but a better understanding of supply chain resilience will feature, along with improved communication’ (Interview F) ‘Each scenario [herein refers to economic condition scenarios] has a set of response functions built in which will trigger switching between models as necessary’ (Interview P) ‘[we] initiated an early planning with agency labour providers, and guaranteed hours for seasonal agency workers to ensure availability of labour’ (Interview AA)

Based on our data analysis and findings, we propose a unique and novel perspective in the investigation of resilience during COVID‐19 by combining dynamic capability and enactment theory. The explanatory power of the combined theoretical lenses provides a richer interpretation of organizational resilience during equivocal environments. A behavioural perspective of sensemaking offers a more nuanced understanding of the construction of dynamic capability phases. Results emphasize that the production and utilization of heuristics to reach resilience during equivocal environments differs between authority‐based and consensus‐based structures. Information and knowledge play an important role in the utilization of risk‐identification heuristics. The vertical communication channels within the authority‐based structures limit the sharing of information and knowledge between peers. To overcome this, authority‐based organizations usually make considerable investments to acquire additional informational support from external bodies. By contrast, consensus‐based structures usually rely on their innate ability to quickly gather information and knowledge locally through internal resources. Therefore, information costs are expected to be lower compared to the authority‐based structures. Furthermore, rules of coordination contribute to the production of different heuristics that are utilized in selecting appropriate actions for the identified risks. Rules of coordination are particularly important for authority‐based structures since they can increase the level of distribution and breadth of knowledge within the organization and reduce the expected information cost. The study's findings corroborate sensemaking research, which suggests that richer group minds facilitate equivocality reduction by having shared overlapping experiences and knowledge (Ellis, Henry and Shockley, 2011; Weick, 2001; Weick, Sutcliffe and Obstfeld, 2005).

Despite the differences concerning information costs, our findings emphasize that both structures use heuristics to identify risks. The sensemaking theory asserts that sensible interpretations of equivocal environments are facilitated through organizational structures and systems (Ellis, Henry and Shockley, 2011; Weick, 2001). Our findings revealed that respondents within authority‐based structures are aware of the limited breadth of information and knowledge associated with vertical information flows within this setting. However, despite limited horizontal information channels within the firm, authority‐based structures emphasize that ‘it creates flexibility for us to rely on our own judgements’ (Interview K), to resort to simple rules (i.e. rudimentary how‐to heuristics), to guide them to select and execute actions. Conversely, consensus‐based structures enjoy a profound information bank through group minds enabling obtainable how‐to heuristics.

The findings of this paper also extend the literature on dynamic capabilities and resilience research, particularly during equivocal environments. While the phase of transformation is found in the dynamic capabilities process of organizations, we further identified a prior step of assessment, an integral step in the process during equivocal environments. Assessment depicts a retention process of objectifying ‘plausible stories’, or actions deployed during the process of seizing. This finding tallies with prior studies that underline the importance of reducing equivocality through acquiring and further developing information for cause–effect relationships, the mitigative actions and consequences of actions, retained for future decisions (Ellis, Henry and Shockley, 2011; Weick, Sutcliffe and Obstfeld, 2005). Overall, we not only contribute to recent calls to incorporate personal attributes of human agents (i.e. risk perceptions) within resilience research (Sarafan, Squire and Brandon‐Jones, 2019; Tangpong, Hung and Li, 2014), but also add to the dearth of empirical investigations of resilience during crises (Duchek, 2020; Linnenluecke, 2017). We provide additional insights into the sensemaking literature by asserting the different types of heuristics produced through different organizational structures.

This study has several implications for practitioners who seek to develop organizational resilience in high environmental uncertainty and equivocality. This study reveals that organizational systems and human bias are the determining factors for managerial ability to perceive and deploy mitigative actions towards SCRs. Consequently, we recommend that companies initiate strategies such as building robust decision support systems, which can strengthen effective communication and interconnectivity, enabling a further reduction of the effect of human biases. Cross‐department training programmes, such as crisis management and business continuity plans, have been mentioned as useful tools for facing uncertainties and increasing the level of available heuristics within firms. We recommend that companies consider a thorough prioritization of critical capabilities and inter‐relationships during the assessment phase, before finally realizing their potential to achieve resilience. Lastly, external assistance such as consultants, trade bodies and audit organizations may also provide useful benchmarks of the organization's views, attitudes and performance against identified risks.

These findings revealed the importance of heuristics during sensemaking through different structures, still unexplored in the literature. Such an interpretation of the results hopefully encourages future research to investigate other aspects that might contribute to such heuristics production. However, we do not suggest that our dichotomization of the two organizational structures represents a company ‘type’ beyond this narrow perspective. It is likely that abundant variables of the organizational structure itself may influence the production of different heuristics and decision‐makers’ approach to the process of sensemaking, along with its contribution to the resilience of organizations. In this regard, our current sample is not equipped to provide such reliable analysis and conclusions. We invite future research to subject our findings to study other elements of organizational structures apart from hierarchies, such as work specifications, unit of command, sphere of control, authority and responsibility, and departmentalization (Robbins and DeCenzo, 2009). In a micro perspective, we further invite scholars to explore detailed individual aspects such as age and culture that might complement the sensemaking process within dynamic capabilities. Moreover, since this research is specifically contextualized for the crisis of COVID‐19 within the UK as a developed country, we invite researchers to conduct further qualitative studies in different settings (e.g. developing countries and other sectors) to bring important nuances into this academic discourse.

## Conclusion

This study develops a framework of organizational resilience to SCRs during equivocal environments that builds on an abductive approach as a starting point to provide insights into different elements of the sensemaking process supporting dynamic capabilities within two different organizational structures. Results reveal important nuances (see Figure 3). In sensing risks related to equivocal environments, both structures utilize risk‐capture heuristics despite higher information costs within authority‐based structures. Concerning seizing, due to a higher level of distribution and breadth of knowledge, consensus‐based structures utilize obtainable how‐to heuristics to select appropriate actions. Conversely, rudimentary how‐to heuristics are utilized by authority‐based structures within this phase. We further identified assessment as an additional step in dynamic capabilities during equivocal environments prior to transformation depicting the process of retention, which emphasizes the importance of cause–effect relationships to enhance future judgements. Nonetheless, as discussed in the contributions and implications section above, further research is required to deepen our understanding of how resilience may differ in equivocal contexts.

## Footnotes

1 While we acknowledge that, in general, the literature on organizational resilience falls into three main categories, for the purpose of our study we explicitly delineate organizational resilience comprising capabilities that are dynamically capable of enhancing organizational processes. Therefore, we incorporate the last category of resistance and recovery (i.e. the ability of organizations to recover *after* disruptions) (Conz and Magnani, 2020; Ducheck, 2020) into our initial adaptation category.
